# Nanotechnology in Targeted Drug Delivery

**DOI:** 10.3390/ijms24098194

**Published:** 2023-05-03

**Authors:** Antonio Di Stefano

**Affiliations:** Department of Pharmacy, University of “G. d’Annunzio” Chieti-Pescara, Via dei Vestini, 66100 Chieti, Italy; antonio.distefano@unich.it

The use of large sized materials in drug delivery raises several challenges, including in vivo stability, poor bioavailability/solubility/absorption, and issues with target-specific delivery, in addition to the side effects of the delivered drugs. Therefore, using new drug delivery systems for targeting drugs to a specific area in the body could be an opportunity to solve these critical issues.

The area of nanotechnology develops nanoscale-sized materials that consist of natural, synthetic/semisynthetic polymers, lipids, or metallic materials. Nanoparticles [NPS] can be used in targeted drug delivery to improve the bioavailability, biodistribution, and accumulation of therapeutics, preferentially in the targeted diseased area, acting as stability enhancers. These colloidal systems can deliver drugs to target sites to improve therapeutic efficiency, reduce toxicity, and reduce side effects, protecting the drug from biological degradation, achieving temporal and spatial control of therapeutics in the specific location of a disease [1,2,3]. The first implementation of nanocarriers for use in drug delivery was based on a passive targeting mechanism that aimed to increase efficiency over traditional free-drug formulations. However, a new approach has been introduced that consists of active targeting through the incorporation of specific ligands to enhance drug delivery to target sites using conjugation strategies or magnetic fields. Hence, nanotechnology has the potential to generate innovation in drug formulations and delivery systems.

Reaching a therapeutic outcome that is able to fight neurodegenerative disorders, tumoral diseases, or immunological disorders is facilitated by an efficient and site-specific delivery of compounds. For these reasons, this Special Issue collates different aspects of research into nanotechnology in order to identify new therapeutic targets and strategies, including review papers [4,5], mathematical models to calculate the trajectories of magnetic NPs in the body or clarify the structures of metal-decorated fullerenes [6,7], the drug delivery systems of different antitumoral agents [8,9,10,11], and the biocompatibilities of stealth liposomes and hybrid nanosystems containing surfactant agents [12,13].

The Editor wishes to thank the researchers who contributed to organize the present issue, who made their knowledge available to the scientific community. I also would like to thank all the Reviewers who have revised the submitted articles; thanks to their deep knowledge of the topics covered, they provided suggestions that have improved the quality of each manuscript reported in this Special Issue.
